# Stress Distribution and Collagen Remodeling of Periodontal Ligament During Orthodontic Tooth Movement

**DOI:** 10.3389/fphar.2019.01263

**Published:** 2019-10-24

**Authors:** Zixin Li, Min Yu, Shanshan Jin, Yu Wang, Rui Luo, Bo Huo, Dawei Liu, Danqing He, Yanheng Zhou, Yan Liu

**Affiliations:** ^1^Laboratory of Biomimetic Nanomaterials, Department of Orthodontics, Peking University School and Hospital of Stomatology, National Engineering Laboratory for Digital and Material Technology of Stomatology, Beijing Key Laboratory of Digital Stomatology, Beijing, China; ^2^Biomechanics Lab, Department of Mechanics, School of Aerospace Engineering, Beijing Institute of Technology, Beijing, China

**Keywords:** extracellular matrix, collagen remodeling, stress distribution, finite element, periodontal ligament, orthodontic tooth movement (OTM)

## Abstract

Periodontal ligament (PDL), as a mechanical connection between the alveolar bone and tooth, plays a pivotal role in force-induced orthodontic tooth movement (OTM). However, how mechanical force controls remodeling of PDL collagenous extracellular matrix (ECM) is largely unknown. Here, we aimed to evaluate the stress distribution and ECM fiber remodeling of PDL during the process of OTM. An experimental tooth movement model was built by ligating a coil spring between the left maxillary first molar and the central incisors. After activating the coil spring for 7 days, the distance of tooth movement was 0.324 ± 0.021 mm. The 3D finite element modeling showed that the PDL stress obviously concentrated at cervical margin of five roots and apical area of the mesial root, and the compression region was distributed at whole apical root and cervical margin of the medial side (normal stress < −0.05 MPa). After force induction, the ECM fibers were disordered and immature collagen III fibers significantly increased, especially in the apical region, which corresponds to the stress concentration and compression area. Furthermore, the osteoclasts and interleukin-1β expression were dramatically increased in the apical region of the force group. Taken together, orthodontic loading could change the stress distribution of PDL and induce a disordered arrangement and remodeling of ECM fibers. These findings provide orthodontists both mechanical and biological evidences that root resorption is prone to occur in the apical area during the process of OTM.

## Introduction

The process of orthodontic tooth movement (OTM) is characterized by collagenous extracellular matrix (ECM) remodeling of bone and periodontal ligament (PDL) mediated by an external mechanical force (Rangiani et al., 2016). After force induction, pressure and tension regions are generated in PDL (Yamaguchi et al., 2010). On the pressure side, disorganized and compressed ECM fibers induce osteoclastic bone resorption, whereas, on the tension side, stretching fibers stimulate osteoblastic bone formation (Oshiro et al., 2010; Ren et al., 2010). It has been shown that the biological response of PDL dependent on mechanical state regulates OTM efficiency (Odagaki et al., 2018). Therefore, it is important to clarify the stress distribution and corresponding biological responses of PDL under an orthodontic force.

During the OTM process, PDL could respond to a mechanical force loading and present two main biological reactions: dynamic changes in collagen content and osteoclastogenesis (Feng et al., 2016). The ECM of PDL is the fibrous connective tissue joining the tooth to its surrounding bone. The collagen fibers with ordered arrangement are the main components. The collagenous ECM is mainly composed of type I (Col-I) and type III (Col-III) collagens, among which Col-I is dominant and mature (Xu et al., 2014). Functionally, Col-I fibers are response for strength and maintain stability of tooth position, whereas Col-III can relieve a tension force on the PDL during OTM (Li et al., 2010). It has been shown that the content of Col-III relative to Col-I increases in the early stage of collagen remodeling (de et al., 2009; Oryan et al., 2010). Another important reaction is osteoclast recruitment in PDL (Xie et al., 2010). It has been shown that PDL cells play crucial roles in osteoclastogenesis by expressing receptor activator of nuclear factor kappa. The recruited osteoclasts resorb and remodel the alveolar bone during OTM (Ha et al., 2003; Hasegawa, 2010).

Unlike bone tissue, the complex and tiny structure of PDL makes it difficult to directly measure the stress distribution in PDL. Lin et al. have predicted that the narrow region of PDL possesses a high strain by using CT images of tooth-PDL-bone specimen under compression (Lin et al., 2014). Recently, a more effective method of numerical simulation by 3D finite element (FE) is applied to directly reveal the stress condition of root (Yan et al., 2013). Here, we firstly used the FE method to evaluate the stress distribution of PDL based on micro-CT images of maxillary first molars under an orthodontic force, and then the biological response of PDL to different stress was analyzed histologically and immunohistochemically.

## Materials and Methods

### Ethics Statement

The animal protocol was approved by the Peking University Ethical Committee (LA2013-92). All efforts were made to minimize animal number and suffering.

### Animal Model of OTM

**Figure 1:**
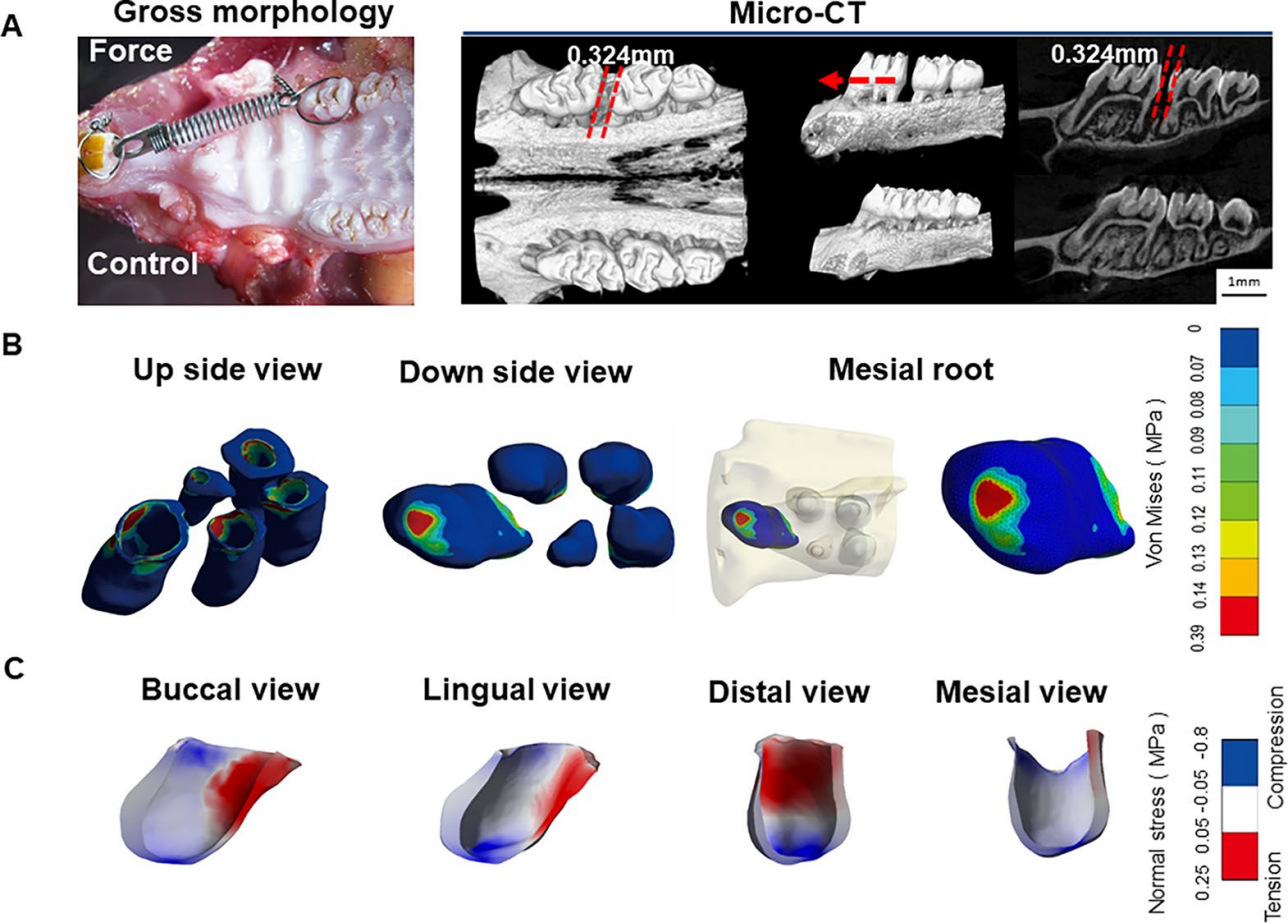
**(A)** Micro-CT images of orthodontic tooth movement (OTM) after 7 days of force application. **(B)** Stress distribution in periodontal ligament (PDL) of maxillary first molars. Blue color represents low stress area (< 0.07 MPa), whereas Von Mises value above 0.07 MPa is set as stress concentration. Stress concentration was in PDL at apical area of the mesial root. **(C)** Tension-compression distribution in PDL of the mesial root. Blue color represents compression area with normal stress < -0.05 MPa while red color shows tension region with normal stress > 0.05 MPa.

Five 6–8-week-old Sprague-Dawley rats were used for building an animal model of experimental tooth movement as previously described (He et al., 2015). Briefly, nickel-titanium coiled springs with 0.2-mm thickness, 1-mm diameter, and 5-mm length (Smart Technology, China) were ligated between the left maxillary first molar and the central incisors of rats and fixed to teeth with 0.2-mm stainless steel wires in the force group. A spring dynamometer device was used to standardize the orthodontic force, and the orthodontic force of the coil spring after activation was approximately 60 g in each rat (Dunn et al., 2007; He et al., 2015). The right side of the same rat was set as a control (**Figure 1A**). After 7 days, all the rats were sacrificed by overanesthesia, and the maxillae were obtained and fixed in 4% paraformaldehyde. A stereo microscope (SWZ1000, Nikon, Japan) was applied to record the occlusal view of each maxilla. The distance of tooth movement was measured between the midpoint of the distal-marginal ridge of the first molar and the midpoint of the mesial-marginal ridge of the second molar (Cao et al., 2014). Every measurement was repeated three times to get the mean value as the final measurement.

### Finite Element Modeling

An accurate model of the maxillary first molar was constructed from the micro-CT images, which were acquired by a Skyscan 1174 micro-CT system (Bruker, Belgium) at resolution of 6.28 μm. The CT images were imported into Mimics software to segment the maxilla by Hounsfield values and manual mask segmentation. Three-dimensional geometry files of maxillae, teeth, and PDL were created for each mask and saved as stereolithographic (STL) files. A Computer Assisted Design software (Geomagic 12.0, Research Triangle Park, NC, USA) was applied to extract surfaces and solids from STL files. Triangle and intersection fixing techniques were performed and then Standard for the Exchange of Product model data files were created and exported separately into ANSYS 18.0 (Canonsburg, PA, USA). To simulate the experimental OTM, line pressure, which was in the position similar to the coil spring, was loaded on the distal surface of the maxillary first molar in the FE model.

### Histological and Collagen Fiber Staining

After micro-CT scanning, the maxillae were demineralized in 10% ethylenediaminetetraacetic acid, dehydrated in ethanol, and embedded in paraffin. Serial longitudinal sections were obtained by vertical cutting of the first molars. Hematoxylin and eosin (HE) staining, Masson’s trichrome staining, and picrocsirius red staining were applied to examine the histochemistry of the samples. HE, Masson’s trichrome, and immunofluorescence staining were used to identify the collagen fiber arrangement. To assess collagen remodeling during OTM, the sections stained with picrosirius red were analyzed *via* a polarizing microscopy, in which collagen type I (Col-I) fibers were red, and collagen type III (Col-III) appeared green. For statistically analysis, we randomly selected three images from each cervical area and central area in the mesial or distal side. As for the apical area, we randomly selected three images from each mesial side and distal side. The proportion of Col-III in the PDL was calculated by an Image-Pro Plus 4.1 software (Media Cybernetics Inc. Silver Springs, MD).

### Immunohistochemical Assay

Immunohistochemistry was performed with a two-step detection kit (Zhongshan Golden Bridge Biotechnology, Beijing, China) as before (Jin et al., 2019). Tissue sections in each group were subjected to antigen retrieval solution, blocked with 5% bovine serum albumin, and incubated overnight with antibodies against the Col-I (1:200; ab34710, Abcam), Col-III (1:200; ab7778, Abcam), and interleukin-1β (IL-1 β, 1:200; ab2105, Abcam). Samples were subsequently incubated with horseradish peroxidase-conjugated secondary antibodies using diaminobenzidine (Zhongshan Golden Bridge Biotechnology, Beijing, China) as chromogen. Three different regions of each side were randomly chosen to count the number of positive cells for statistical analysis.

### Tartrate-Resistant Acid Phosphatase Staining

The sections were deparaffinized to perform tartrate-resistant acid phosphatase (TRAP) test using a leukocyte acid phosphatase kit (387A, Sigma) according to the manufacturer’s protocol. TRAP-positive multinucleated (> 3 nuclei) cells that are attached to the alveolar bone surface mesial to the distal buccal roots were counted.

### Statistical Analysis

All the data were expressed as mean ± SD. All statistical analyses were performed with a GraphPad Prism 6 software (GraphPad Software, San Diego, CA, United States) and *P* < 0.05 was considered to be statistically significant. Furthermore, t-test was used to evaluate the difference between groups in different regions.

## Results

### Stress Distribution in PDL Under an Orthodontic Force

Tooth movement through bone is induced by an appropriate mechanical force. Activation of coil springs could generate a light orthodontic force about 60 g, which made the left first molar move to the mesial about 0.324 ± 0.021 mm after 7 days. This distance is consistent with that reported in the literatures (Dunn et al., 2007; He et al., 2015). In contrast, the right first molar, which served as the control, did not move. The micro-CT images further confirmed the tooth movement without obvious root resorption under the light force (**Figure 1A**).

Based on the micro-CT images, a 3D FE model of five roots of the first molar during OTM was developed. This model could accurately reproduce the tooth-PDL-bone structure, which is generally assumed to be a simple geometry in previous FE analyses (Kamble et al., 2012; Zhang et al., 2017). In the FE analysis, we mainly focused on horizontal force by the line pressure, mimicking orthodontic tipping tooth movement. The PDL stress (> 0.07 MPa) obviously concentrated at cervical margin of the mesial side of five roots from the top and apical area of the mesial root from the bottom. In contrast, low stress (< 0.07 MPa) is distributed in the middle of five roots and the apical regions of the other four roots, except for the mesial root (**Figure 1B**).

Normal stress field was further applied to assess the tension-compression area. From the tension-compression analysis of the mesial root, the compression region was distributed at whole apical root and cervical margin of the medial side (normal stress < -0.05 MPa), whereas the tension zone was present at two thirds of crown of the distal side (normal stress > 0.05 MPa) (**Figure 1C**). This finding is different from the classical OTM theory that symmetric compression (mesial), and tension (distal) areas are present in PDL. However, a previous FE analysis also demonstrates that no distinct pressure and tension regions are detected for complex mechanical properties of PDL (Cattaneo et al., 2010). The stress concentration on the apical region might be closely correlated to high incidence of root resorption during OTM.

### The Orthodontic Force Increased Col-III Expression in PDL

**Figure 2:**
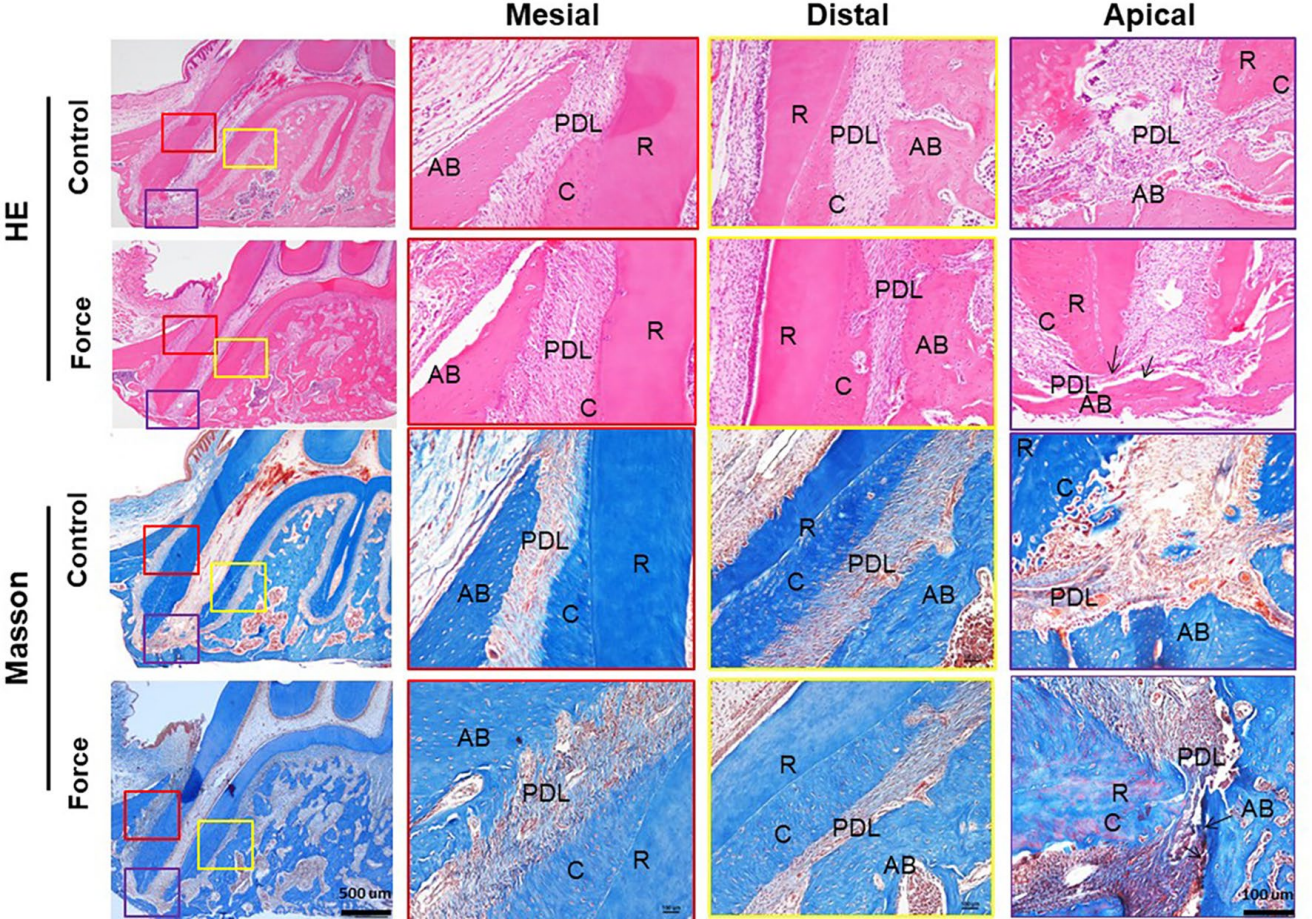
HE and Masson stainings of PDL of the mesial root. The PDL fibers in the mesial area were irregular and the apical PDL was broken in the force group (arrows). AB, alveolar bone; R, root; PDL, periodontal ligament; C, cementum.

**Figure 3:**
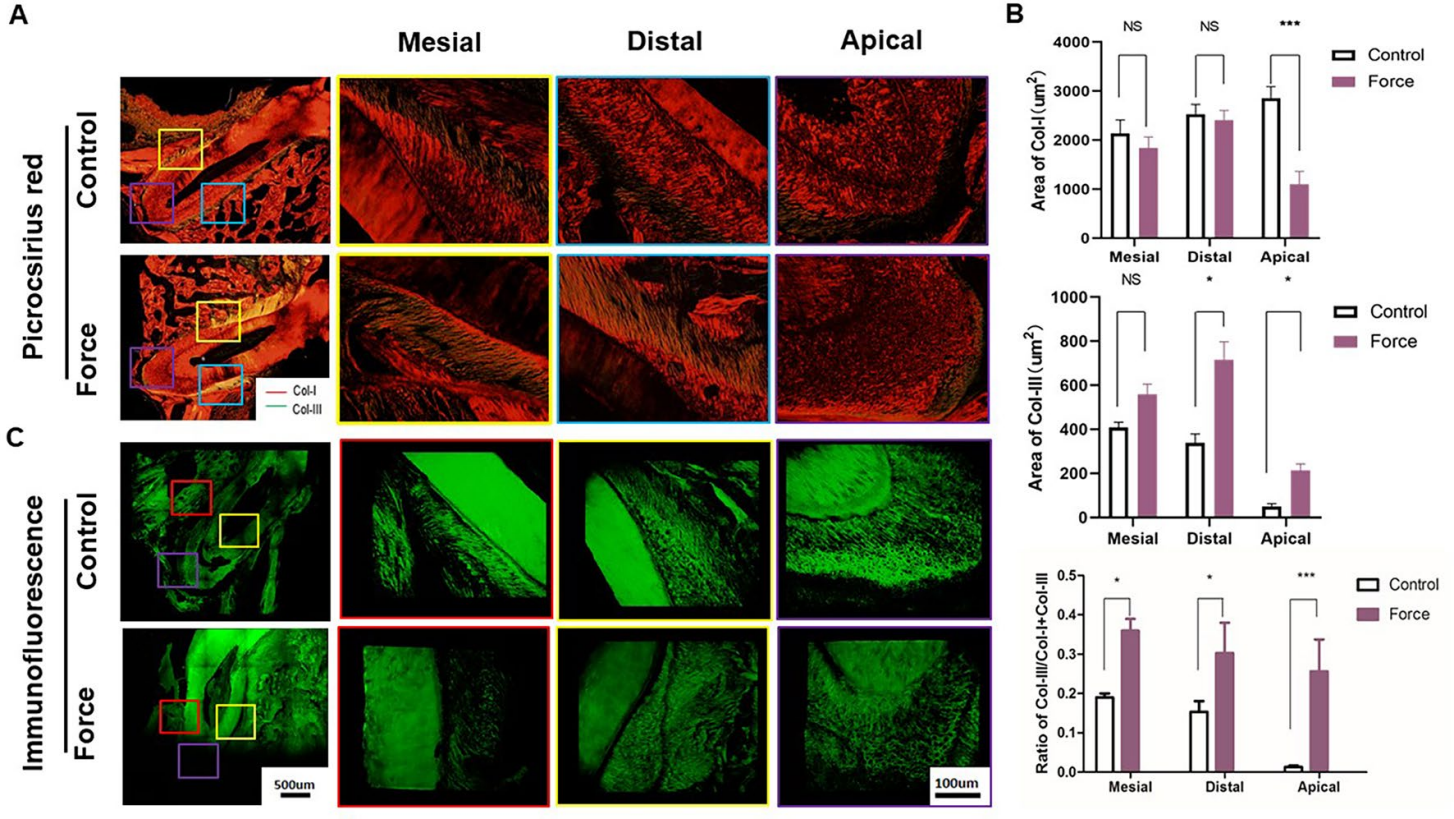
**(A)** Picrocsirius red staining of periodontal ligament (PDL). Col-I fibers stain red, whereas Col-III fibers are green stained. **(B)** Semiquantification of Col-I and Col-III fiber area in **(A)**. There were more Col-III fibers in the distal and apical regions and less Col-I fibers in the apical regions in the force group (n = 18, mean ± SD). *: P < 0.05, ***: P < 0.001, NS, not significant. **(C)** Immunofluorescence staining of PDL showing disoriented fibers in the force group.

Collagen fibers in an ordered arrangement are the main components of ECM in PDL. Most fibers at the angle of 45° to the root are parallel with each other. Based on the FE results, we focused on the collagen remodeling of the mesial root. From HE and Masson stainings, the PDL fibers in the mesial area were irregular in shape and the cementum was discontinuous in the force group. Especially, the apical PDL was disoriented and even broken in the force group. In contrast, the periodontal space was well maintained between the root and alveolar bone, and the contour of the cementum was continuous in the control group (**Figure 2**). This finding was further confirmed by picrosirius red and immunofluorescence stainings (**Figure 3**).

**Figure 4:**
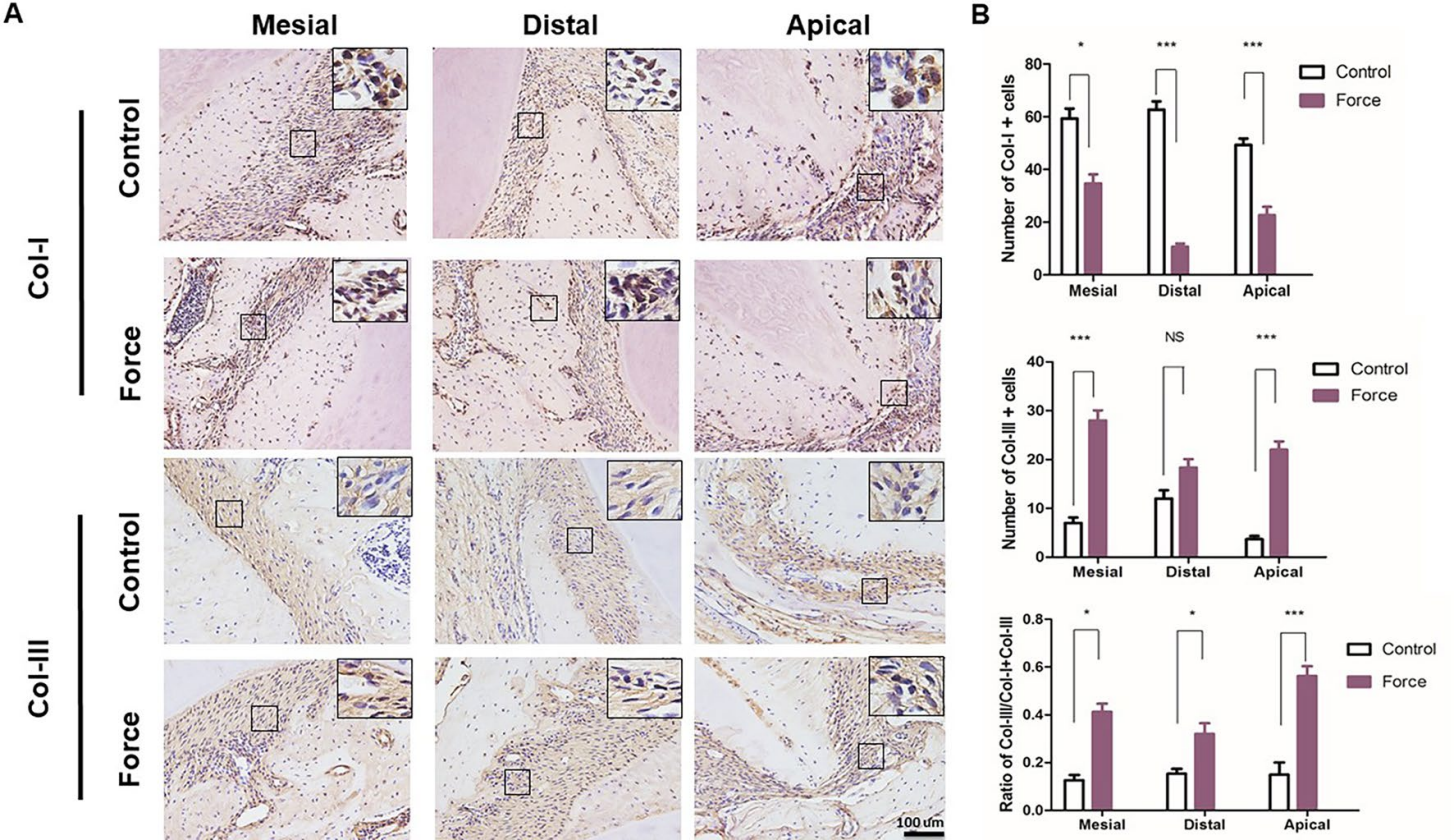
**(A)** Immunohistochemical staining of Col-I and Col-III. **(B)** Semiquantification of Col-I^+^ and Col-III^+^ cells in **(A)**. There were more Col-III^+^ and less Col-I^+^ cells in the mesial and apical regions in the force group (n = 18, mean ± SD). *: *P* < 0.05, ***: *P* < 0.001, NS, not significant.

**Figure 3** illustrated that red-stained Col-I bundles were dominant in the control group. Compared to the apical area, there were much more green-stained Col-III fibers in the mesial and distal areas without loading. After force induction, the Col-III expression was significantly enhanced compared with the control group. This trend was more obvious in the apical region (p < 0.001) (**Figure 3B**). The immunofluorescence staining further revealed the disordered arrangement of collagen fibers in PDL in the force group. From immunohistochemistry, more Col-I positive staining cells were found in the control group compared with the force group. On the contrary, the opposite trend was observed in the Col-III positive staining cells. The ratio of Col-III positive cells was significantly increased in the force group (p < 0.05), especially in the apical region (p < 0.001) (**Figure 4**). Taken together, these results indicate that the immature collagen fibers increase during OTM.

### Osteoclast Recruitment in PDL Under an Orthodontic Force

**Figure 5:**
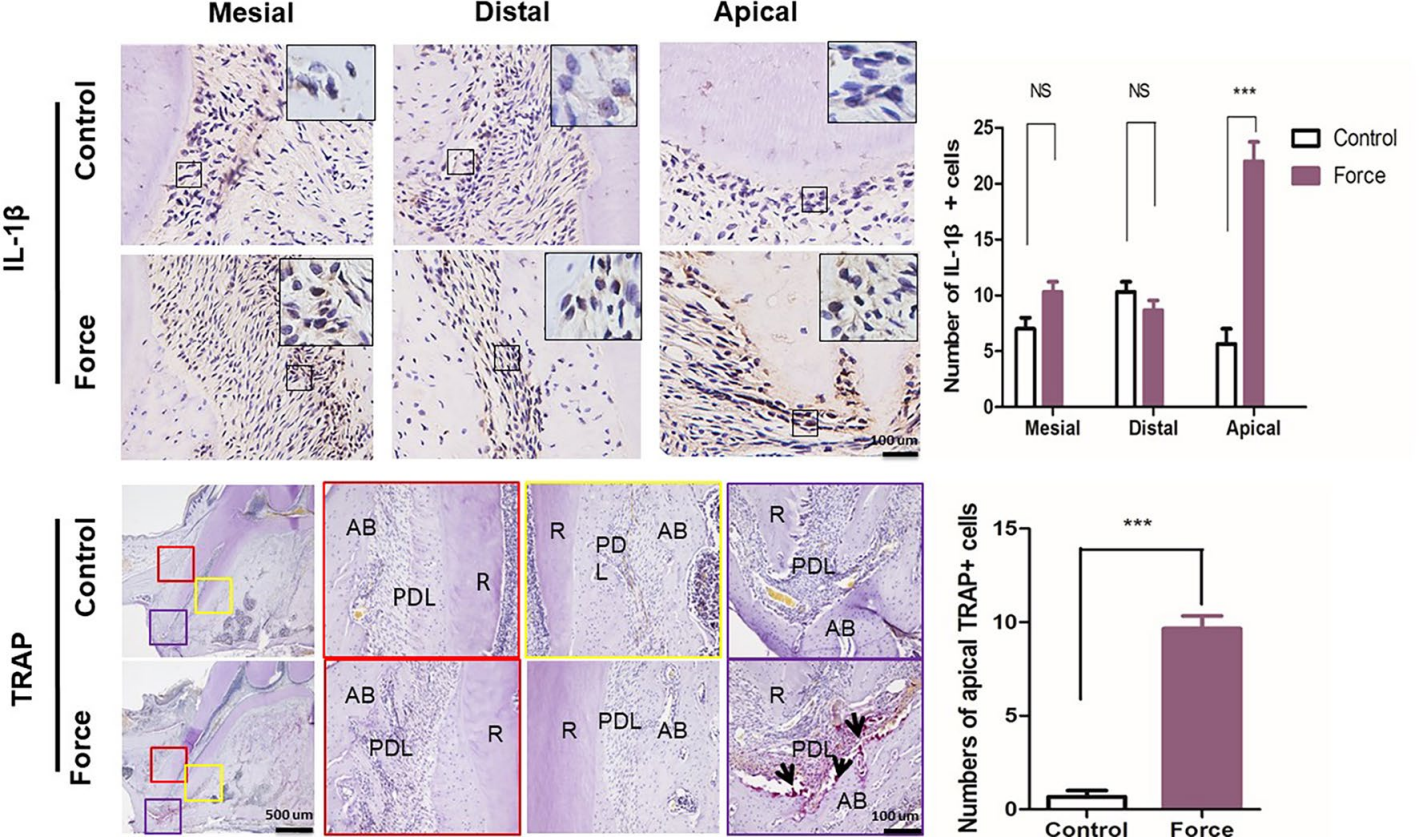
**(A)** Immunohistochemistry and semiquantification of IL-1β positive cells (n = 18, mean ± SD). ***: *P* < 0.001, NS: not significant. **(B)** Tartrate-resistant acid phosphatase (TRAP) staining of the mesial root. Semiquantification of TRAP^+^ cells indicated that the force group had much more TRAP^+^ cells than the control group (n = 18, mean ± SD). ***: *P* < 0.001. R, root; PDL, periodontal ligament; AB, alveolar bone. Arrows, osteoclasts.

The sustained force could change the chemical environment by releasing inflammatory cytokines and, therefore, influence bone and root resorption. Immunohistochemical analysis demonstrated that an orthodontic force could dramatically enhance the IL-1β expression level especially in the apical region. The release of IL-1β could induce osteoclast differentiation and further promote root resorption (Kim et al., 2009; Baba et al., 2011) (**Figure 5A**). We next examined whether the force-induced osteoclastogenesis was influenced in different areas of the mesial root during the process of OTM. The TRAP staining showed that the number of osteoclasts was significantly increased in the apical region in the force group compared with the control group. No positive staining was found in the mesial and distal regions in both the control and the force groups (**Figure 5B**).

## Discussion

Orthodontic tooth movement is a synergistic result of physical phenomenon and biological responses of the tooth-alveolus complex to an externally applied force (Kalajzic et al., 2014). During the OTM process, PDL responds to mechanical force stimulation and provides a microenvironment for cellular reactions and tissue remodeling. The remodeling of collagenous ECM fiber in PDL corresponding to mechanical loading is largely unknown, although the bone remodeling process during OTM is well investigated (Rangiani et al., 2016). In the present study, we first accurately demonstrated PDL stress concentration regions by a 3D FE modeling and then particularly analyzed biological responses of PDL including collagen fiber remodeling and osteoclast recruitment corresponding to stress distribution. We found that a light orthodontic force temporarily broke collagen orderly arrangement and increased immature Col-III fiber number and inflammation of PDL, especially in the apical region, which corresponds to stress concentration area. Although orthodontic root resorption is a common and well-known phenomenon, the direct mechanical evidence is lacking. Here, a 3D FE modeling combined with histological analyses provides orthodontists both mechanical and biological evidences that root resorption is prone to occur in the apical area during the process of OTM.

Orthodontic treatment is highly related to collagen fiber remodeling in the ECM of PDL, which mainly consists of mature Col-III and immature Col-I fibers (Becker et al., 1991; Xu et al., 2017). Therefore, investigations on dynamic changes of collagen content would help us to clarify the biological response of PDL to mechanical loading. In our study, enhanced expression of Col-III in the tension areas of PDL, especially in the apical region, indicated active remodeling of PDL under orthodontic loading. In contrast, Col-I expression decreased significantly in the force group. This finding is consistent to a previous report that the Col-III/Col-I ratio is increased in the early phase of collagen remodeling, especially under a tension force. The accumulation of Col-III might contribute to relieving the tension force placed on PDL during OTM. During the late phase of PDL ECM remodeling, Col-III could be gradually replaced by Col-I until a normal Col-III/Col-I ratio is obtained. Stress from orthodontic loading is transmitted from PDL ECM *via* integrins, which induce a change in ECM synthesis, PDL remodeling, and ultimately tooth movement (Krishnan and Davidovitch, 2006; Ma et al., 2017). It has been shown that diabetes enhance the Col-III/Col-I ratio and prolong the PDL remodeling process under an orthodontic force (Li et al., 2010). Mechanistically, diabetes could elevate matrix metalloproteinase levels, which rapidly degrade collagen in PDL ECM, disturb fibroblast function, and finally complicate OTM (Chang et al., 2008).

Osteoclastogenesis in PDL is another key process during OTM, in which the recruited osteoclasts resorb and remodel the alveolar bone (Li et al., 2010; Hou et al., 2014). Osteoclasts are characterized by high expression levels of TRAP, osteoprotegerin, and Cathepsin K (Gerogianni et al., 2005; Rangiani et al., 2016). Here, we showed that a light orthodontic force enhanced the number of TRAP^+^ osteoclasts in the apical region corresponding to stress concentration area. This suggests that bone or root resorption is prone to occur at the apical region, which is highly consistent with clinical phenomena (Ling et al., 2010). At the early stage of remodeling, no obvious bone or tooth resorption occurred under a continuous light force for 7 days in the study. According to the report of Wellington et al, osteoclasts induced by an orthodontic force originate by the fusion of recently recruited preosteoclasts from the marrow instead of from local PDL cells, although there are osteoclasts residing in the PDL space (Rody et al., 2001).

Mechanical forces cause capillary vasodilatation, followed by migration of leukocytes and the release of cytokines (Sasano et al., 2010). Several studies have provided experimental evidence to support a statement that cytokines regulate the bone and PDL remodeling processes during OTM (Norevall et al., 2010; Rangiani et al., 2016; Tsuge et al., 2016). Among the cytokines, IL-1β is thought to play a prominent role during OTM. Blocking IL-1β by a soluble receptor inhibits tooth movement (Lages et al., 2009; Baba et al., 2011). Various studies have shown that IL-1β stimulates bone resorption and inhibits bone formation *in vivo* (Nguyen et al., 1991; Baba et al., 2011; Diercke et al., 2012). The observation that transient IL-1β elevation in alveolar bone precedes the increase in osteoclasts population in several days suggests that recruitment of new preosteoclasts may be important in OTM.

As the highlight of our study, we investigated from the perspective of mechanics with modified 3D FE modeling and had directly proven that the stress distribution of apical region was more special compared with mesial and distal regions, which also provided orthodontists direct evidence that root resorption was prone to occur in the apical area.

## Conclusion

Orthodontic loading could change the stress distribution of PDL and induce a disordered organization and remodeling of collagen fibers in the ECM of PDL. Immature collagen III fibers and inflammation increased during OTM, especially in the apical region, which corresponds to stress concentration area. Further research is needed to translate biological concepts into clinical practice.

## Data Availability Statement

All datasets generated for this study are included in the manuscript/supplementary files.

## Ethics Statement

The animal study was reviewed and approved by Peking University Ethical Committee (LA2013-92).

## Author Contributions

YL, YZ, and DH designed the study, analyzed the data, and revised the manuscript. ZL and MY performed the experiments, interpreted the data outcomes, and drafted the manuscript. SJ, YW, RL, BH, and DL contributed to the experimentation and data analysis. All authors reviewed and approved the final manuscript.

## Funding

This work was financially supported by the Projects of Beijing Nova Programme No. Z171100001117018 (YL), the Beijing Nova Programme Interdisciplinary Cooperation Project No. Z181100006218135 (YL), and the National Natural Science Foundations of China No. 81871492 (YL), No. 81571815 (YL) and No. 81600893 (DH).

## Conflict of Interest

The authors declare that the research was conducted in the absence of any commercial or financial relationships that could be construed as a potential conflict of interest.
